# Supplementary material for: EnzML: multi-label prediction of enzyme classes using InterPro signatures
Source: BMC Bioinformatics. 2012 Apr 25;13:61. doi: 10.1186/1471-2105-13-61 (PMC3483700; doi:10.1186/1471-2105-13-61)
Supplement: Addtional file 5 — The Java code to format the data files, evaluate and predict. The file enzml_java_code.tar.gz contains the Java code used to format database data to ARFF and XML formats, to execute cross and train-test (jackknife) evaluations and to record evaluation results to database. More information is included in the readme.txt file and the Javadoc files. The code can be used with a MySQL database. To use a different database software, other JDBC drivers might be required. [file 1471-2105-13-61-S5.gz › java_code/enzml2011/doc/uk/ac/ed/inf/taweka/weka/AttributeFactory.html]

AttributeFactory


---


|  |  |  |
| --- | --- | --- |
| **Overview** \| **Package** \| **Class** \| **Use** \| **Tree** \| **Deprecated** \| **Index** \| **Help** | |  |
| **PREV CLASS**   **NEXT CLASS** | **FRAMES**    **NO FRAMES**     **All Classes** |  |
| SUMMARY: NESTED \| FIELD \| CONSTR \| METHOD | DETAIL: FIELD \| CONSTR \| METHOD |  |


---


## uk.ac.ed.inf.taweka.weka Class AttributeFactory

```
java.lang.Object
  uk.ac.ed.inf.taweka.weka.AttributeFactory
```

**Direct Known Subclasses:**: MulanAttributeFactory

---

``` public class AttributeFactory extends java.lang.Object ```

The attribute factory produces binary and nominal Weka attributes.
A binary attribute is a special nominal attribute with only 2 values: yes/no
(or present/absent or zero/one). The attribute name often represents the
characteristic that can be present or absent. Example: if the attribute name
is a protein domain, the value yes/no represents whether the domain is
present or absent in that protein sequence (instance).
A nominal attribute is an attribute with a set of (more than 2) possible
string values.

**Version:**
:   20 Feb 2008

**Author:**
:   Luna De Ferrari luna.deferrari-at-ed.ac.uk

---

| **Field Summary** | |
| --- | --- |
| `static java.lang.String` | `ABSENT_OPTION` |
| `static java.lang.String` | `PRESENT_OPTION` |


**Constructor Summary**

`AttributeFactory(DataSetGenerator dataSetGenerator)`
  
          Takes the attribute names from the dataset generator object.


| **Method Summary** | |
| --- | --- |
| `weka.core.Attribute` | `generateBinaryAttribute(java.lang.String attributeName)`             Generates a binary attribute |
| `weka.core.Attribute` | `generateNominalAttribute(java.lang.String attributeName, java.util.Vector<java.lang.String> values)`             Generate a nominal attribute (with a list of possible values). |
| `weka.core.Attribute` | `generateNominalAttributeWithDummy(java.lang.String attributeName, java.util.Set<java.lang.String> classValues)`             Generate a nominal attribute (with a list of possible values). |
| `java.lang.String` | `getAttributeAbsentOption()` |
| `weka.core.Attribute` | `getAttributeByName(java.lang.String name)` |
| `java.lang.String` | `getAttributePresentOption()` |
| `java.util.Vector<weka.core.Attribute>` | `getAttributes()` |
| `java.util.TreeSet<java.lang.String>` | `getAttributesNames()` |
| `java.util.Vector<java.lang.String>` | `getBinaryAttributeValuesSet()`             Get the possible attribute values for a yes/no attribute. |
| `weka.core.Attribute` | `getClassAttribute()`             Get last attribute as class attribute |
| `DataSetGenerator` | `getDataSetGenerator()` |

**Methods inherited from class java.lang.Object**

`equals, getClass, hashCode, notify, notifyAll, toString, wait, wait, wait`

**Field Detail**

### ABSENT\_OPTION

```
public static final java.lang.String ABSENT_OPTION
```

**See Also:**: Constant Field Values

---


### PRESENT\_OPTION

```
public static final java.lang.String PRESENT_OPTION
```

**See Also:**: Constant Field Values


**Constructor Detail**

### AttributeFactory

```
public AttributeFactory(DataSetGenerator dataSetGenerator)
```

:   Takes the attribute names from the dataset generator object.

    **Parameters:**: `dataSetGenerator` -


**Method Detail**

### generateBinaryAttribute

```
public weka.core.Attribute generateBinaryAttribute(java.lang.String attributeName)
```

:   Generates a binary attribute

    :   **Parameters:**: `attributeName` - the attribute name **Returns:**: the weka attribute

---


### generateNominalAttribute

```
public weka.core.Attribute generateNominalAttribute(java.lang.String attributeName,
                                                    java.util.Vector<java.lang.String> values)
```

:   Generate a nominal attribute (with a list of possible values).

    :   **Parameters:**: `attributeName` - the attribute name **Returns:**: the weka attribute

---


### generateNominalAttributeWithDummy

```
public weka.core.Attribute generateNominalAttributeWithDummy(java.lang.String attributeName,
                                                             java.util.Set<java.lang.String> classValues)
```

:   Generate a nominal attribute (with a list of possible values). Inserts a
    dummy value as first element to solve the problem at
    http://weka.wikispaces.com/ARFF+%28stable+version%29

    :   **Parameters:**: `attributeName` - the attribute name **Returns:**: the weka attribute

---


### getAttributeAbsentOption

```
public java.lang.String getAttributeAbsentOption()
```

---


### getAttributeByName

```
public weka.core.Attribute getAttributeByName(java.lang.String name)
```

---


### getAttributePresentOption

```
public java.lang.String getAttributePresentOption()
```

---


### getAttributes

```
public java.util.Vector<weka.core.Attribute> getAttributes()
```

---


### getAttributesNames

```
public java.util.TreeSet<java.lang.String> getAttributesNames()
```

---


### getBinaryAttributeValuesSet

```
public java.util.Vector<java.lang.String> getBinaryAttributeValuesSet()
```

:   Get the possible attribute values for a yes/no attribute. Beware!: the
    option "no" has to be listed as first option for the sparse ARFF format
    to work! http://weka.wikispaces.com/ARFF+%28stable+version%29

    :   **Returns:**: The vector with the two values, for example: ["no", "yes"].

---


### getClassAttribute

```
public weka.core.Attribute getClassAttribute()
```

:   Get last attribute as class attribute

    :   **Returns:**: a Weka `Attribute`

---


### getDataSetGenerator

```
public DataSetGenerator getDataSetGenerator()
```


---


|  |  |  |
| --- | --- | --- |
| **Overview** \| **Package** \| **Class** \| **Use** \| **Tree** \| **Deprecated** \| **Index** \| **Help** | |  |
| **PREV CLASS**   **NEXT CLASS** | **FRAMES**    **NO FRAMES**     **All Classes** |  |
| SUMMARY: NESTED \| FIELD \| CONSTR \| METHOD | DETAIL: FIELD \| CONSTR \| METHOD |  |


---
